# Benign Adenomyoepithelioma of the Breast: A Case Report

**DOI:** 10.7759/cureus.60801

**Published:** 2024-05-21

**Authors:** Smaran S Teru, Dawn Cox, Brynn Wolff

**Affiliations:** 1 Medicine, Lake Erie College of Osteopathic Medicine, Erie, USA; 2 Breast Surgery, University of Pittsburgh Medical Center, Harrisburg, USA

**Keywords:** ductal carcinoma in situ, self-breast examination, breast cancer prognosis, partial mastectomy, breast adenomyoepithelioma

## Abstract

Adenomyoepitheliomas of the breast are rare tumors that are characterized histologically as having both epithelial and myoepithelial components. While adenomyoepitheliomas are considered benign lesions, existing literature supports their potential for malignant transformation. These tumors also exhibit nonspecific and variable findings on noninvasive imaging, posing additional challenges in management. We present a rare case of an adenomyoepithelioma diagnosed in a 65-year-old female who was treated with surgical resection of her tumor, with histopathology negative for malignant transformation. By describing this patient’s management course, we aim to contribute to existing literature analyzing adenomyoepitheliomas and help guide future treatment.

## Introduction

Adenomyoepitheliomas (AMEs) are rare tumors, histologically featuring a bicellular pattern consisting of epithelial and myoepithelial cells [1]. Although most AMEs are benign lesions, one or both cellular components of the tumor may exhibit malignant transformation, characterized by increased mitotic rates, infiltrative growth patterns, cytologic atypia, and/or necrosis [2]. A case series published by Parikh et al. emphasizes the diagnostic challenges imposed by AMEs and highlights the necessity of surgical excision for definitive histopathologic diagnosis and exclusion of malignancy [3]. Here, we present a rare case of a 65-year-old female who underwent lumpectomy after being diagnosed with benign AME using core needle biopsy.

## Case presentation

A 65-year-old female with a relevant past medical history of stage 1A ER/PR-negative, HER2-positive microinvasive ductal carcinoma in situ (DCIS) of the upper outer left breast presented for further evaluation of a recently diagnosed AME of her right breast. In 2014, the patient underwent a left partial mastectomy and left sentinel lymph node biopsy. Given the size and pathology of her lesion, she did not receive adjuvant chemotherapy or HER2-targeted therapy, but she did complete adjuvant radiation therapy.

**Figure 1:**
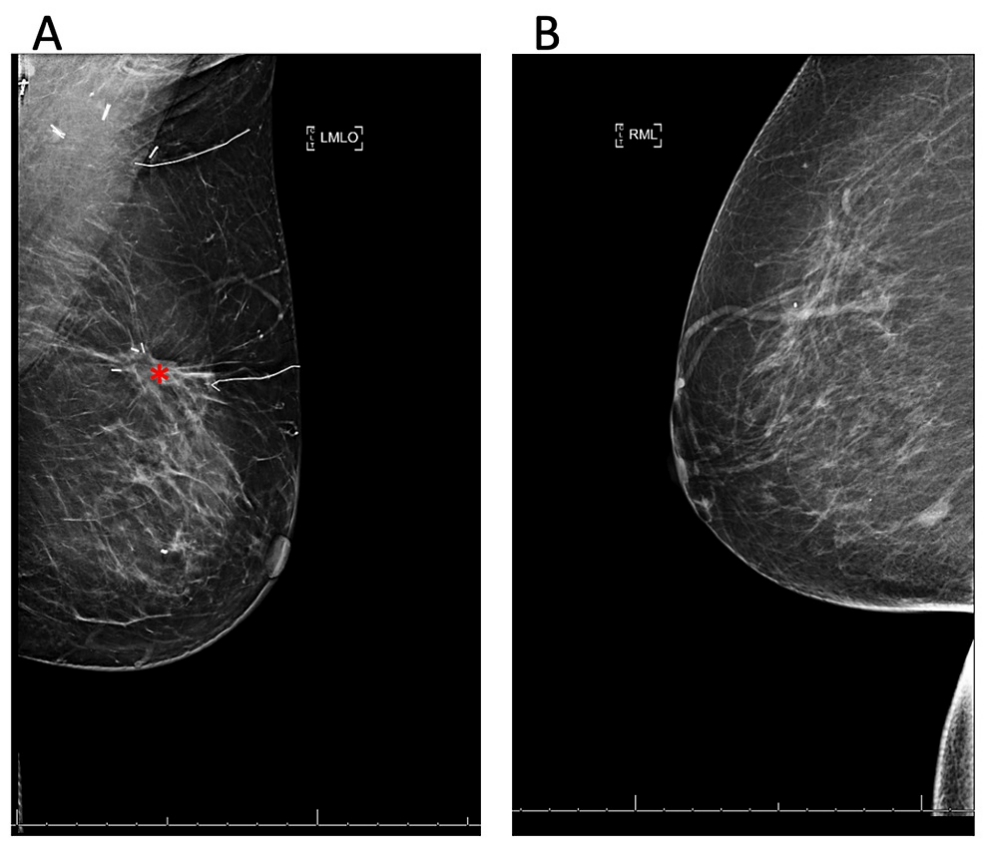
Bilateral screening mammogram of the left breast (A) and right breast (B). (A) An irregular 2.3 x 1.4 x 1.7 cm mass is seen with shadowing and blood flow marked by the red asterisk corresponding to the area of indentation and palpable abnormality in the 2-3 o'clock position, 6 cm from the nipple at the previous site of the lumpectomy in the left breast. (B) Slightly increasing punctuate and pleomorphic calcifications are seen in a grouped distribution with an associated asymmetry in the lower inner quadrant of the right breast at 9 o'clock.

**Figure 2:**
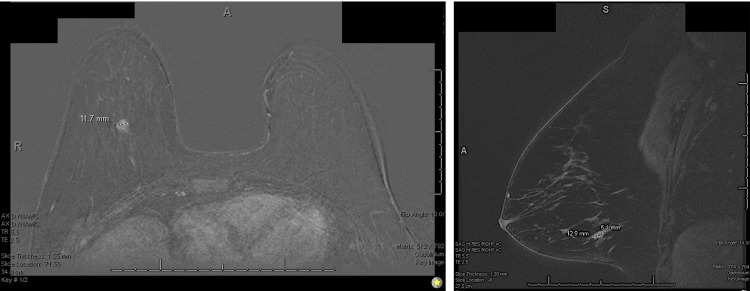
Bilateral breast MRI demonstrating tumor dimensions. T2-weighted images demonstrate a T2 hyperintense biopsy marker abutting the inferior margin of a mildly T2 hyperintense 12 x 13 x 5 mm rapidly enhancing mass at 6 o'clock indicated in the dotted line, middle to posterior depth of the right breast. No other rapidly enhancing or suspicious abnormalities were identified. No lymphadenopathy was identified in the right axilla.

In November 2022, the patient underwent a diagnostic bilateral mammogram, with no concerning findings for new masses or malignancy. In August of 2023, the patient underwent a repeat diagnostic bilateral mammogram after noticing increasing density and dimpling at the surgical site of her left breast. The results of the diagnostic bilateral mammogram were significant for a new cluster of calcifications in the left lower inner breast and an avascular, hypoechoic mass with increasing density in the right breast (Figure 1). Given her new imaging findings in both breasts, an ultrasound-guided biopsy and HydroMARK™ clipping of both breast masses was performed to rule out the recurrence of her previously diagnosed cancer. Ultrasound-guided biopsy of her right breast mass revealed findings of an AME with no associated atypia or necrosis, and an ultrasound-guided biopsy of her left breast mass revealed benign stromal fibrosis. After careful consideration, the patient decided to pursue a right breast ultrasound-guided, needle-localized partial mastectomy, given the tumor’s potential for malignant transformation. Pre-operative magnetic resonance imaging (MRI) images are shown in Figure 2. The tumor and an additional superior margin were successfully removed, with an intraoperative radiograph showing the surgical clip within the excised tumor. Gross morphology of the resected tumor revealed a well-circumscribed, solid tan mass measuring 1.0 x 1.0 x 0.9 cm. Tissue pathology of the tumor showed concordant findings with her previously diagnosed benign AME, and tissue pathology of the superior margin revealed ductal hyperplasia, margins negative for tumor and atypia, with focal calcifications and staining positive for p63 and SMM on immunohistochemistry. The patient was advised to return to the breast oncology clinic for continued breast examinations and surveillance imaging, as directed by her breast surgeon.

## Discussion

AMEs are a rare subtype of breast tumors. The World Health Organization Classification of Breast Tumors in 2012 further characterized AMEs as benign or malignant, arising from epithelial, myoepithelial, or both components [4,5]. Due to the tumor’s rarity, literature to guide clinical decision-making is limited. However, retrospective case review series have been published, which help further characterize AMEs. A case series by Moritz et al. included 14 patients diagnosed with an AME with a mean age of 51 years, of which three had previous or synchronous malignancy [6]. Approximately 20% of patients diagnosed with an AME have a concurrent or prior malignancy, according to a retrospective study of 12 patients with histologically confirmed AME [7]. Additionally, there are case reports demonstrating synchronous DCIS or invasive ductal carcinoma with AMEs [8-12]. However, histologic derivations of one lesion from the other have not been established [8]. Further research to investigate a potential relationship between epithelial malignancy and AMEs might enhance the understanding of its pathophysiology.

Parikh et al. describe that the initial diagnosis of an AME with imaging and core biopsy has been shown to pose challenges [3]. On mammography, seven cases of benign and malignant AME exhibited nonspecific and variable findings ranging from masses and focal asymmetries to microcalcifications, consistent with previous literature [3]. On ultrasound, the seven cases demonstrated an oval-shaped mass with indistinct margins and heterogeneous echotexture, consistent with previous literature reporting features of oval or irregular hypoechoic masses with circumscribed or micro-lobulated margins [3,7,13]. On MRI, three of the seven reported cases exhibited heterogeneous enhancement with varying shapes and margins, consistent with previous literature reporting round to irregularly shaped masses with smooth, indistinct, or irregular margins and heterogenous enhancement [3,14,15]. As a result, variable diagnostic imaging features pose challenges to diagnosing and differentiating AME from other lesions. Therefore, histopathology with core needle biopsy is used for the initial diagnosis despite additional variability. Parikh et al. reported three initial core biopsy results of sclerosing adenosis and nodular sclerosis that were eventually found to be benign AME on surgical excision [3]. The bicellular components of AMEs create additional challenges due to their morphological variability, often displaying a variety of patterns and metaplasia [3,16,17].

This case emphasizes the necessity to investigate nonspecific imaging findings in the setting of previous breast malignancy. The patient presented with no dermatological manifestations or symptoms in her right breast but with a past medical history of microinvasive DCIS. Bilateral ultrasound-guided core needle biopsies were performed to rule out malignant recurrence of initially diagnosed cancer. A biopsy of the right breast mass revealed a biphasic lesion composed of epithelial and myoepithelial cells, with the myoepithelial component staining positive for p63 and SMM on immunohistochemistry. However, the features differentiating an AME as benign or malignant are not well-defined but include cytological atypia, nuclear pleomorphism, cellular necrosis, and increased mitotic rates [18]. Moreover, immunohistochemistry is rarely used to differentiate benign and malignant AME as myoepithelial expressions are maintained in malignant tumors [19]. Therefore, the decision to surgically excise the patient’s tumor and the superior margin was performed conservatively to avoid local recurrence or possible malignant transformation. As described by Parikh et al., local recurrence or distant metastasis was not identified in the seven patients, six of whom elected for surgical excision of their AME [3]. Therefore, surgical excision remains the recommended diagnostic and therapeutic intervention for rare cases of AMEs despite limited reported literature.

## Conclusions

AMEs of the breast are rare tumors that present diagnostic challenges given their variable presentation using noninvasive imaging. Ultimately, the diagnosis of AME is established with a core needle biopsy and the resultant display of bicellular components and positively staining immunohistochemistry. Benign AMEs confirmed histologically should be excised to prevent the risk of potential malignant transformation, especially in the setting of prior breast malignancy. This case highlights that AMEs should be included in the differential for symptomatic or asymptomatic breast masses. Their variable presentation, transformation, and recurrence urge clinicians to investigate and treat these lesions to improve patient outcomes promptly. Research examining the tumor’s pathogenesis and long-term response to wide surgical excision is needed to help guide future management.
